# Proteomic Evaluation of Neonatal Exposure to 2,2′,4,4′,5-Pentabromodiphenyl Ether

**DOI:** 10.1289/ehp.8419

**Published:** 2005-10-06

**Authors:** Henrik Alm, Birger Scholz, Celia Fischer, Kim Kultima, Henrik Viberg, Per Eriksson, Lennart Dencker, Michael Stigson

**Affiliations:** 1 Department of Pharmaceutical Biosciences, Division of Toxicology, and; 2 Department of Environmental Toxicology, Uppsala University, Sweden

**Keywords:** 2D-GE, brain development, brain growth spurt, MALDI–ToF–MS, neonatal, neurodegeneration, PBDE-99, PKC, proteomics

## Abstract

Exposure to the brominated flame retardant 2,2′,4,4′,5-pentabromodiphenyl ether (PBDE-99) during the brain growth spurt disrupts normal brain development in mice and results in disturbed spontaneous behavior in adulthood. The neurodevelopmental toxicity of PBDE-99 has been reported to affect the cholinergic and catecholaminergic systems. In this study we use a proteomics approach to study the early effect of PBDE-99 in two distinct regions of the neonatal mouse brain, the striatum and the hippocampus. A single oral dose of PBDE-99 (12 mg/kg body weight) or vehicle was administered to male NMRI mice on neonatal day 10, and the striatum and the hippocampus were isolated. Using two-dimensional fluorescence difference gel electrophoresis (2D-DIGE), we found 40 and 56 protein spots with significantly (*p* < 0.01) altered levels in the striatum and the hippocampus, respectively. We used matrix-assisted laser desorption ionization time-of-flight mass spectrometry (MALDI–ToF–MS) to determine the protein identity of 11 spots from the striatum and 10 from the hippocampus. We found that the levels of proteins involved in neurodegeneration and neuroplasticity (e.g., Gap-43/neuromodulin, stathmin) were typically altered in the striatum, and proteins involved in metabolism and energy production [e.g., α-enolase; γ-enolase; ATP synthase, H^+^ transporting, mitochondrial F_1_ complex, β subunit (Atp5b); and α-synuclein] were typically altered in the hippocampus. Interestingly, many of the identified proteins have been linked to protein kinase C signaling. In conclusion, we identify responses to early exposure to PBDE-99 that could contribute to persistent neurotoxic effects. This study also shows the usefulness of proteomics to identify potential biomarkers of developmental neurotoxicity of organohalogen compounds.

The importance of pre- and postnatal development for the onset of diseases later in life is recognized but not well understood. Thus, assessing fetal and infant exposure to environmental neurotoxicants and the developmental implications may provide important insights into the pathogenesis of neurodegenerative diseases. Studies in mice have identified a window of critical vulnerability in neonatal brain development during which persistent neuro-toxic effects may be induced by a diverse array of xenobiotics, ranging from pharmacologic substances (e.g., ketamine; Fredriksson et al. 2004) to environmental organohalogens [e.g., 1,1-bis(4-chlorophenyl)-2,2,2-trichloroethane (DDT), polychlorinated biphenyls (PCBs), and polybrominated diphenyl ethers (PBDEs); Eriksson 1997; Eriksson et al. 2001; Eriksson and Talts 2000; Viberg et al. 2002, 2003a, 2003b]. This critical developmental phase coincides with the period of brain growth spurt (BGS), which is characterized by a number of neurodevelopmental changes, including dendritic and axonal outgrowth, establishment of neural connections, and synaptogenesis (Davison and Dobbings 1968). For the PBDEs, different neurotransmitter systems have been suggested as potential targets for the neurotoxic effects (Mariussen and Fonnum 2003; Meerts et al. 2004; Viberg et al. 2002, 2003a, 2004b), even if the mechanisms by which these systems are damaged during the BGS are unclear.

Human exposure to PBDEs is of growing concern worldwide. For example, PBDE levels have increased significantly in the breast milk of Swedish women over the past three decades (Noren and Meironyte 2000). The trend in the United States is the same (Schecter et al. 2005) but with levels of PBDEs in blood and adipose tissue lipid that are 10–100 times those in human tissues in Europe (Schecter et al. 2003). The reason for this difference is not clear. One of the most common PBDE congeners found in human and environmental samples (Darnerud et al. 2001) is PBDE-99, which has been shown to impair spontaneous behavior and habituation capability in adult mice after neonatal exposure to relatively moderate doses (Viberg et al. 2002). In addition the effects appear to worsen with increasing age (Viberg et al. 2004a). It remains to be defined how such late responses to early exposure may reflect the long-term consequences of brain development being disrupted during sensitive stages. Here we present a proteomics approach toward understanding the primary damaging events that occur in the developing brain during PBDE-99 exposure and toward finding potential biomarkers of developmental neurotoxicity for this group of compounds. We report protein level changes in the striatum and in the hippocampus, both of which are part of the developing cholinergic and catecholaminergic systems that have been implicated as targets for some PBDEs (Mariussen and Fonnum 2003; Meerts et al. 2004; Viberg et al. 2002, 2003a, 2004b). This finding suggests neurodegeneration and aberrant neuroplasticity as possible causes of injury.

## Materials and Methods

### Animals.

We purchased pregnant NMRI mice from B&K (Sollentuna, Sweden). Mice were individually housed in plastic cages at 22°C ambient temperature with a 12/12-hr light/dark cycle, and supplied *ad libitum* with standardized pellet food (Lactamin, Stockholm, Sweden) and tap water. Cages were inspected for newborn pups twice a day (0800 and 1800 hr), and found pups were assigned day 0. Litter size was adjusted to 10–12 pups within 24 hr of birth by random culling. Litters containing pups of both sexes were housed with their mothers up to an age of 4 weeks. The animals were treated humanely and with regard for alleviation of suffering; animal experiments were conducted in accordance with and after approval from the local ethical committee (Uppsala University and Agricultural Research Council) and by the Swedish Animal Welfare Agency (license 2003/87).

### Treatments and sample collection.

The polybrominated diphenyl ether PBDE-99 (2,2′,4,4′,5-pentabromodiphenyl ether), donated by Å. Bergman (Department of Environmental Chemisty, Stockholm University, Stockholm, Sweden), was dissolved in a 1:10 mixture of egg lecithin (Merck, Darmstadt, Germany) and peanut oil (*Oleum arachidis*), and sonicated with water to obtain a 20% (w/w) water:fat emulsion containing 1.2 mg/mL PBDE-99. On postnatal day 10, mice (three to four litters) were given 10 mL/kg body weight (bw) of this emulsion as a single oral dose (12 mg/kg bw PBDE-99) via a metal gastric tube; control mice (three to four litters) received vehicle only. Animals were sacrificed by cervical dislocation 24 hr after treatment. The brains were rapidly dissected out, and the hippocampus and the striatum from each brain were isolated, rapidly put on ice, and stored at −70°C until further processing. The entire procedure from sacrifice to when each brain region was put on ice was completed within 3 min for each mouse.

### Sample preparation.

**Figure 1:**
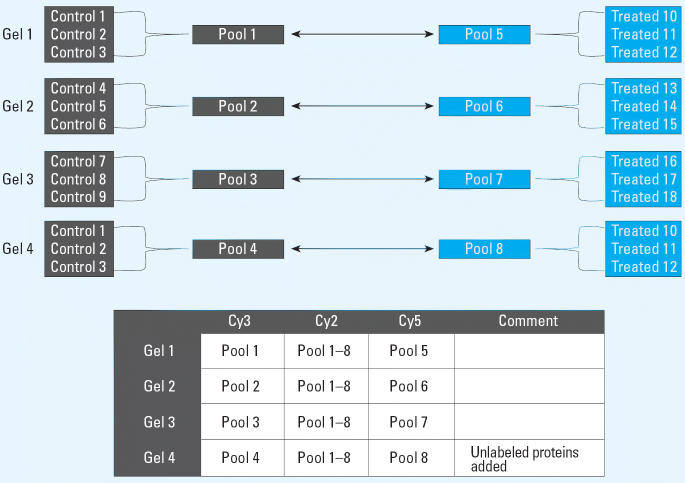
Experimental design and pooling procedure. Individual control (1–9) and treated (10–18) tissue samples were separately pooled into three control (1–3) and treated (5–7) pools per sample type (striatum and hippocampus). In addition to these biological replicates, we made a technical replicate of pools 1 and 5 (4 and 8, respectively). Control pools (1–4) were labeled with Cy3, treated pools (5–8) with Cy5, and a mixture of all pools (pools 1–8) with Cy2 as internal standard. Four gels were run in parallel, as indicated, with extra unlabeled protein added to gel 4.

We separately pooled (to reduce variance from individual variation) control and treated samples from the striatum (~ 160–180 mg wet weight each) and the hippocampus (~ 250–270 mg wet weight each), as illustrated in Figure 1. Frozen tissue from three pups in each pool was lysed with 4 volumes of 9.6 M urea, 4% 3-[(3-cholamidopropyl) dimethyl-ammonio]-1-propanesulfonate (CHAPS), 70 mM dithiothreitol (DTT); 5% immobilized pH gradient (IPG) buffer, pH 3–10, and homogenized with a sonicator (Branson Sonifier cell disruptor B15; Kebo-Grave, Stockholm, Sweden) in an Eppendorf tube on ice in pulses of 10 sec, followed by ultracentrifugation for 1 hr at 100,000 × *g* (Ultra centrikon; Kontron, Neufarn, Germany). We collected and cleaned supernatants from lipids and nucleic acids using the 2D Clean-up Kit (GE Healthcare, Uppsala, Sweden) according to the manufacturer’s instructions. The total protein concentration of each sample was determined using the 2D Quant Kit (GE Healthcare) according to the manufacturer’s protocol. To minimize protease activity, we performed the entire procedure on ice whenever possible.

### 2D-DIGE.

For two-dimensional fluorescence difference gel electrophoresis (2D-DIGE), we labeled 50 μg each of control, treated, and internal standard protein sample with cyanine dye (Cy)3, Cy5 and Cy2, respectively, according to the manufacturer’s descriptions for CyDye DIGE Fluor minimal dyes (GE Healthcare). The internal standard was a mixture of equal amounts of protein from all pools (Figure 1). Four 2D-DIGE gels each for the striatum and the hippocampus (i.e., a total of eight gels) were run in parallel. Two of the gels (gels 4 and 8; Figure 1) were technical replicates with 150 μg unlabeled protein from a pool of control animals added to allow for protein identification (see below) directly from a 2D-DIGE gel. Before the first-dimension isoelectric focusing (IEF), a 50-μg aliquot from each of the three labeling mixes (see above) was combined with rehydration buffer [containing 7 M urea, 4% (w/v) CHAPS, 1% (w/v) DTT, and 0.5% (v/v) Pharmalytes (GE Healthcare) that covered the pH interval (pH 4–7) of the IPG strips], to give a final volume of 450 μL. In-gel rehydration of the 24-cm IPG strips (GE Healthcare) with the 450-μL rehydration buffer (including the protein sample), was performed at room temperature in the dark for 12 hr according to the manufacturer’s instructions (GE Healthcare). IEF was run on an IPGPhor (GE Healthcare) at 500 V for 1 hr, at 1 kV for 1 hr, and at 8 kV until a total of 64 kVh was reached. After IEF, the strips were equilibrated for 2 × 15 min by gentle shaking in a buffer containing 50 mM Tris–HCl (pH 6.8), 6 M urea, and 2% sodium dodecyl sulfate (SDS), supplemented with 2% DTT in the first equilibration step and 2.5% iodoacetamide in the second. For the second dimension SDS–polyacrylamide gel electrophoresis (SDS–PAGE), the equilibrated strips were put on top of precast large-format 12.5% polyacrylamide gels (GE Healthcare) and were run using an Ettan DALTsix large-format vertical system (GE Healthcare). The gels were run at 5 W for 45 min before increasing to 11 W per gel until the bromophenol blue dye front had reached the bottom of the gel. The temperature was kept constant at 27°C. The gels were scanned with a Typhoon 9400 fluorescence scanner (GE Healthcare).

### Image analysis and statistics.

The fluorescent images of the 2D-DIGE gels were analyzed using the DeCyder software suite (GE Healthcare) with default settings. The Cy2, Cy3 and Cy5 images for each gel were merged in the Differential In-gel Analysis DeCyder module, and spot boundaries were automatically detected. Spots containing dust particles and other nonproteinaceous artifacts were flagged manually and excluded from further analysis. We matched spots from the different gels using the automatic matching function in the Biological Variance Analysis DeCyder module after prior landmark settings and confirmed manually by comparing each spot against the master gel. The normalized volumes for Cy3 and Cy5 were exported from the DyCyder software and analyzed in the R computing environment from the GNU Project web server (http://www.r-project.org/) using the limma package (Bioconductor Software Project, Cambridge, MA, USA; http://www.bioconductor.org; Smyth and Speed 2003). Normalized volume refers to the volume normalized across the three dyes and across the gels.

To determine differential expression of proteins, we used the moderated *t*-statistics as used, as previously implemented by Smyth (Smyth and Speed 2003). For the statistical analysis, we used the log_2_ of the normalized volumes. We used only proteins present (localized) across all four gels, thereby allowing for no missing values. For each gel and spot, the ratio Cy5/Cy3 was calculated, and ratios less than unity were folded over by inverting the ratio value. The upper 95^th^ percentile of these ratios was taken as a practical measure of a ratio above which real effects could be expected (Tonge et al. 2001). We then calculated the log_2_ 95% ratios for each gel. To predict a value above which the absolute spot ratio for a future gel is likely to signify a real effect and not be caused merely by chance, we calculated one limit for the striatum and another one for the hippocampus as follows:


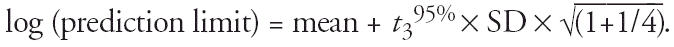


The t_3_^95%^ is the upper 95% point of the student’s *t*-distribution with 3 degrees of freedom because four gels were used to estimate the SD of the log 95% ratios, and the mean is the average of the 95% ratios (Tonge et al. 2001).

### Protein identification.

After the image analysis, gels containing the additional load of proteins unlabeled proteins (gel 4; Figure 1) were stained with Colloidal Coomassie Brilliant Blue G (Acros Organics, Geel, Belgium) and matched to the fluorescent 2D-DIGE images. We picked selected spots (see “Results”) using a spot-picking instrument made in-house and stored the gel plugs (with a diameter of 2 mm) in Eppendorf tubes at −70°C until analysis. We also picked some spots from a preparative gel with a protein load of 400 μg that covered the same pH range as the 2D-DIGE gels and run similarly. For peptide mass fingerprinting, proteins were digested with trypsin according to Havlis et al. (2003). Briefly, gel plugs were washed twice in 100 μL 50 mM ammonium bicarbonate/50% methanol for 30 min followed by an additional wash in 75% acetonitrile (ACN) for 10 min. Plugs were dried followed by in-gel digestion using 10 μL (200 ng) trypsin in 20 mM ammonium bicarbonate for 60 min at 37°C. Peptides were extracted with 50% ACN/0.1% trifluoroacetic acid (TFA, completely dried, and resuspended in 50% ACN/0.1% TFA. Before adding the peptide samples to plates for matrix-assisted laser desorption ionization time-of-flight mass spectrometry (MALDI–ToF–MS), the α-cyano-4-hydroxycinnamic acid (4-HCCA) matrix was made saturated in 50% ACN/0.1% TFA, diluted 1:2, spotted onto plates, and dried. MALDI–ToF–MS was performed at the Wallenberg Center North Expression Proteomics Facility (Uppsala University, Uppsala, Sweden) using a Bruker Ultraflex II (Bruker Daltonics, Billerica, MA, USA). The peptide masses were matched with the theoretical peptide masses of all proteins in the National Center for Biotechnology Information (NCBI) nonredundant database (http://www.ncbi.nih.gov) using the MASCOT search engine (http://www.matrixscience.com/; Perkins et al. 1999). Monoisotopic masses and a mass tolerance of 0.5 Da was used throughout. We confirmed further the protein identity of selected spots (see “Results”) using MALDI–MS/MS analysis, using Bruker Ultraflex II.

### Western blot.

To simultaneously detect Gap-43 and the endogenous reference protein β-tubulin, we used rabbit anti-Gap-43 antibody (Santa Cruz Biotechnology Inc., Santa Cruz, CA, USA) and mouse anti-β-tubulin antibody (Sigma Chemical Co., St. Louis, MO, USA), respectively, with the ECL Plex Western Blotting Detection System (GE Healthcare) according to the manufacturer’s instructions

## Results

**Figure 2:**
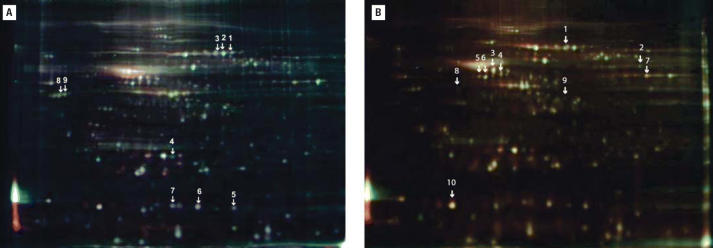
2D-DIGE image of fluorescently labeled proteins from striatum (*A*) and hippocampus (*B*) of PD11 mice. Proteins were extracted from the striatum (*A*) and hippocampus (*B*) of mice 24 hr after they received a single oral dose of PBDE-99 (treated) or vehicle (control) on PD10. Proteins were labeled with Cy3 (control), Cy5 (treated), and Cy2 (mixture of control and treated as internal reference; see “Material and Methods”) and separated on pH 4–7 IPG strips in the first dimension. This procedure was followed by separation on 12.5% SDS–polyacrylamide gels in the second dimension, and the gels were scanned with a Typhoon 9400 fluorescent scanner. The arrows point to the 9 (*A*) and 10 (*B*) spots found to be regulated and identified spots, respectively. The numbers correspond to the numbers in Table 1.

**Figure 3:**
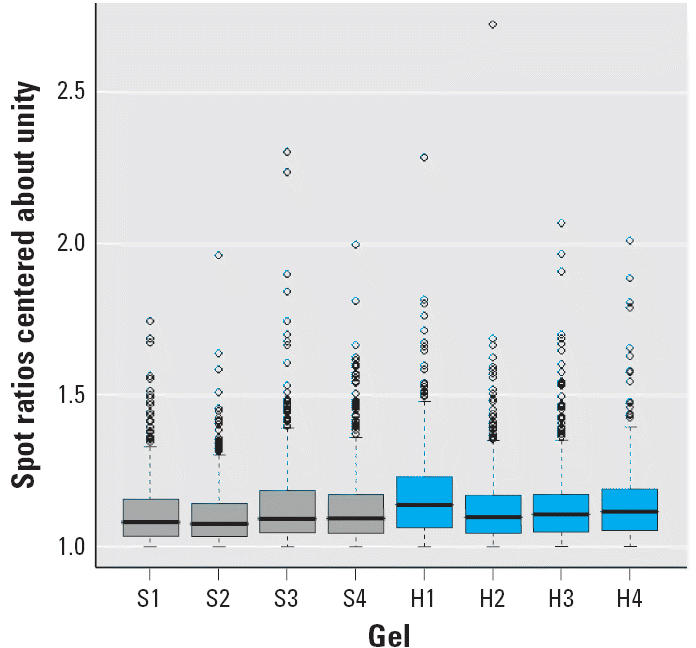
Box plots of the spot ratios from the four gels from the (*S*) striatum and the (*H*) hippocampus. The box limits are the first and third quartile, closely representing 50% of the data. The whiskers extend to the most extreme data point, which is no more than 1.5 times the length of the box away from the box. The overall average and SD of the log_2_ 95% ratios averages 0.141 ± 0.016 for the striatum and 0.165 ± 0.022 for the hippocampus, corresponding to log_2_ 95% prediction limits of 0.199 and 0.242, respectively. S1–S4, gels 1–4 (see Figure 1) for striatum; H1–H4, gels 1–4 for hippocampus.

**Figure 4:**
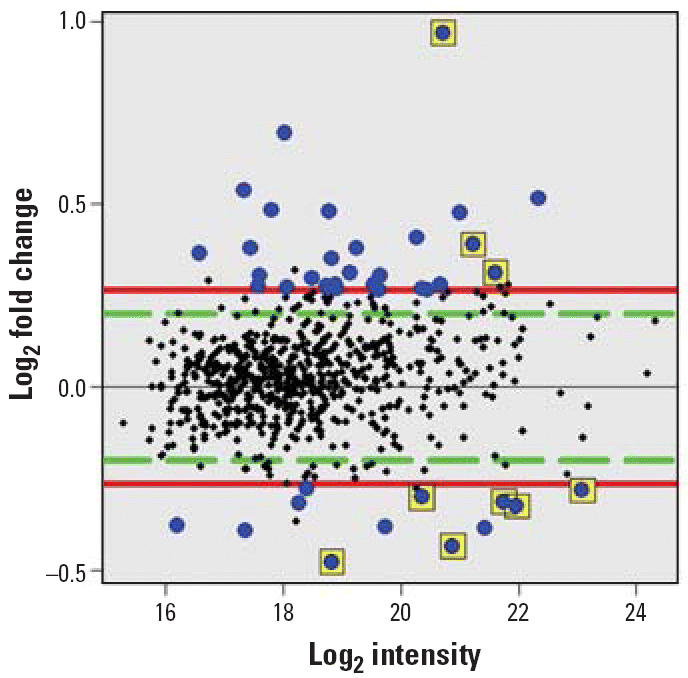
Measurement of relative expression versus average log intensity (MA) plot showing the result of the statistical analysis of the striatum proteins. The log_2_ values of spot intensity on the *y*-axis are plotted against log_2_ values of fold change on the *x*-axis. The intensity was calculated as the log_2_ of the raw intensity of the average of Cy3 and Cy5 across all four gels. The spots correspond to the striatum proteins identified on all four DIGE gels. The spots marked in blue represent the differently regulated proteins, whereas the squared blue spots represent the nine proteins picked and identified from the striatum gel 4.

**Figure 5:**
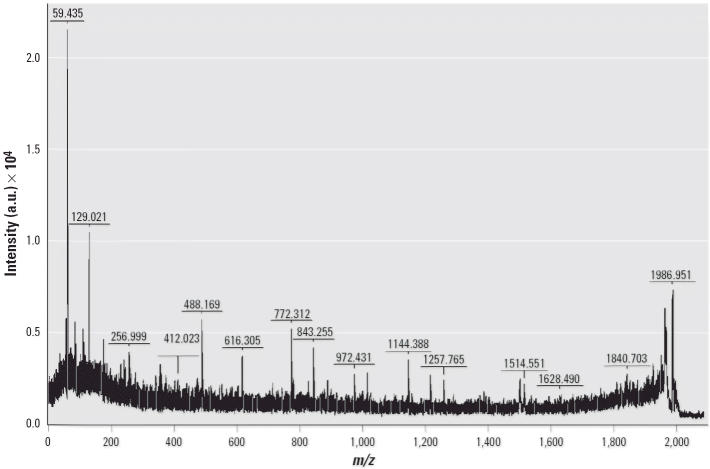
MS/MS identification of Gap-43 (neuromodulin). MS/MS spectrum for spot 10 in Figure 2A. The parent ions *m/z* 1986.91 and 1217.56 were used for MS/MS identification.

**Figure 6:**
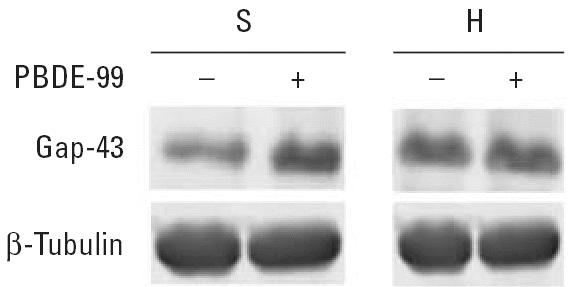
Western blot validation of Gap-43 expression. Gap-43 and the endogenous reference protein β-tublin were simultaneously detected in control (−) and PBDE-99–exposed (+) striatum (S) and hippocampus (H) samples after SDS–PAGE and electroblotting to nitrocellulose.

To explore protein changes associated with developmental neurotoxicity of PBDE-99, we used a previously established mouse model in which a single oral dose of PBDE-99 (12 mg/kg bw) administered during the neonatal period (day 10) induces persistent abnormalities in adult spontaneous behavior, learning, and memory (Eriksson et al. 2001; Viberg et al. 2004b). The striatum and the hippocampus from treated and control animals, respectively, were separately pooled and subjected to replicated 2D-DIGE analysis (Figure 1), addressing both biological and technical variation. To allow for protein identification directly from the 2D-DIGE gels, one of the four replicate gels (gel 4, Figure 1) was a technical replicate loaded with additional unlabeled protein (150 μg) from a control animal. This increased protein load had no apparent adverse effect on the fluorescent spot pattern, which was highly similar between gels regardless of sample type (Figure 2), or on the quantitative analysis, as indicated by the reduced variance when including all four replicate gels in the analysis (data not shown). The average number of spots found in the gels from the striatum and the hippocampus was 1,893 ± 216 and 1,616 ± 185, respectively, where the error is the standard error of the mean. Only the 685 and 651 spots found in all four gels from the striatum and the hippocampus, respectively, were used for further analysis. We determined spots to be differentially expressed by the following three criteria: *a*) the log_2_ fold change (treated/control) had to be > 95% prediction limit (0.199 for the striatum and 0.242 for the hippocampus; Figure 3) in three of four gels; *b*) the average log_2_ fold change had to be > 0.263 (corresponding to a 1.2-fold up- or down-regulation); and *c*) the difference had to be statistically significant (*p* < 0.01). This set of criteria resulted in 40 statistically differentially expressed spots in the striatum (Figure 4) and 56 in the hippocampus (not shown), corresponding to 5.8 and 8.6% of the spots identified on all four gels, respectively. We selected spots for protein identification based on spot intensity (Figure 4) and spot integrity (Figure 2). Using MALDI–ToF–MS, we determined the protein identity (Table 1) for 9 spots from the striatum (Figure 2A) and for 10 spots from the hippocampus (Figure 2B), respectively. These spots were found to correspond to six (striatum) and nine (hippocampus) protein identities, respectively, only one of which (mortalin) was common to both brain structures (Table 1). Among the proteins identified from the striatum, two (represented by 3 spots) were up-regulated and four (represented by 6 spots) were down-regulated by PBDE-99 exposure, whereas all proteins identified from the hippocampus were up-regulated (Table 1). The 3 well-separated spots (5, 6, and 7; Figure 2A) in the striatum, identified as stathmin (Table 1), indicate the existence of three protein variants (all down-regulated) of similar molecular weight but different charge. This finding may imply alternative posttranslational modification or isoform expression of this protein, which may also be the case for the 2 juxtaposed but distinctively separate spots (8 and 9; Figure 2A) in the striatum identified as Gap-43 (neuromodulin), and for spots 5 and 6 (Figure 2B) in the hippocampus, identified as γ-enolase (Table 1), respectively. The identity of spot 8 in the striatum, 1 of the 2 juxtaposed spots (8 and 9; Figure 2A) identified as Gap-43 (Table 1) in a separate preparative gel (not shown), was further confirmed by MALDI–MS/MS (Figure 5). The increased level of Gap-43 in the striatum but not in the hippocampus after PBDE-99 treatment was confirmed by Western blot (Figure 6).

## Discussion

Neurotoxicity of PBDEs in the neonatal mouse has been shown ultimately to target the developing cholinergic and catecholaminergic systems (Mariussen and Fonnum 2003; Meerts et al. 2004; Viberg et al. 2002, 2003a, 2004b). However, the cellular events from the period of BGS to permanent impairment of adult spontaneous behavior and memory functions (Eriksson et al. 2001) are not fully understood. We used 2D-DIGE, a powerful proteomics approach for identifying quantitative protein differences between two samples (Tonge et al. 2001), to show that a single dose of PBDE-99 in neonatal mice induces relatively rapid (within 24 hr of exposure) changes in the levels of several proteins in two critical brain regions of the developing cholinergic and monoaminergic systems, the striatum and the hippocampus (Table 1). It has been shown earlier that neonatal PBDE-99 exposure in this mouse model is associated with a 1.2-fold decrease in the density of cholinergic nicotinic receptors in the hippocampus at an adult age (Viberg et al. 2004b). In the present study, we identify various other proteins that change > 1.2-fold with statistical significance (*p* < 0.01) in these brain regions neonatally. Even if 2D gel electrophoresis may be unrivaled among protein separation techniques in terms of resolution, its major limitation is lack of sensitivity (Yarmush and Jayaraman 2002). Hence, the fluorescent spots discernible in Figure 2 represent only a few of the most abundantly expressed proteins in each brain structure. In other words, the proteins for which we detect significantly altered levels shortly after exposure to PBDE-99 (Table 1) are likely to be major cellular components. Moreover, the highly similar fluorescent spot patterns in gels separating proteins from the striatum (Figure 2A) and the hippocampus (Figure 2B) indicate that both of these brain structures may express essentially the same major proteins on postnatal day (PD) 11, thereby showing their functional importance under normal conditions of BGS. Any altered levels of such proteins, being either a cause or a consequence of cellular events initiated by PBDE-99 action, would be expected to have effects on or reflect processes related to neural development and brain maturation.

Neurite sprouting and outgrowth are among the critical processes occurring during the BGS period. We found PBDE-99 to induce significantly increased levels of neuromodulin (Gap-43), and decreased levels of stathmin in the striatum (Table 1) but not in the hippocampus. The expression of neuromodulin and stathmin closely correlates with neurite outgrowth (Grenningloh et al. 2004; Skene 1989). The expression of neuromodulin is nerve cell–specific and developmentally regulated (Oestreicher et al. 1997). It has become evident that neuromodulin plays key roles in guiding the growth of axons and in modulating the formation of new connections (Benowitz and Routtenberg 1997), as in reinduction of axonal growth for regeneration after damage (Skene 1989). Similar changes (i.e., up-regulation of neuromodulin and down-regulation of stathmin) have been observed in neurodegenerative disorders such as Alzheimer disease (Cheon et al. 2001; Rekart et al. 2004), most likely reflecting compensatory sprouting.

Interestingly, both neuromodulin and stathmin are protein kinase C (PKC) substrates (Duraj et al. 1995; Iannazzo 2001). The role of PKC signaling in neuronal development and function as well as in learning and memory processes has been intensely studied (Birnbaum et al. 2004; Rekart et al. 2005), and PKC isoforms are among the few proteins known to be affected by PBDE-99 (Kodavanti and Ward 2005; Madia et al. 2004). For example, PBDE-99 has been reported to induce activation of PKC isoforms α, ɛ, and ζ in astrocytoma cells *in vitro* (Madia et al. 2004). In primary cultures of rat cerebellar granule neurons, PBDE-71 and PCBs stimulate the release of arachidonic acid (Kodavanti and Derr-Yellin 2002), a *cis*-unsaturated free fatty acid that activates PKC-ɛ (Yaguchi et al. 2005), through a mechanism dependent on phospholipase A_2_ (PLA_2_) activity (Nishizuka 1995).

In the hippocampus of animals exposed to PBDE-99, we found increased levels of three metabolic proteins previously reported to be components of PKC-ɛ signaling complexes (Edmondson et al. 2002): α-enolase; ATP synthase, H^+^ transporting mitochondrial F_1_ complex, β subunit (Atp5b); and isocitrate dehydrogenase 3 (NAD^+^) α (Idh3a) (Table 1). We also detected an increase of γ-enolase in the hippocampus (Table 1) and an increase, although not statistically significant (data not shown), of α-enolase in the striatum. Heterodimers of α- and γ-enolases have been observed in rat brain synaptic terminals (Ueta et al. 2004), but any co-regulation of these proteins in the mouse hippocampus in response to PBDE-99 has yet to be determined.

Several lines of evidence thus point toward a possible role for PKC signaling as a proximate target for the neurotoxicity of PBDE-99. PKC activity has also been reported to be affected by other environmentally relevant xenobiotics such as dioxins (Kim and Yang 2005), PCBs (Tilson and Kodavanti 1998), and tetrabromo-bisphenol A (Reistad et al. 2005).

It is well established that the brain is vulnerable to exogenous insults during the BGS period (Eriksson 1997; Ikonomidou et al. 2000; Olney 2004). Also, an accentuation of the naturally occurring apoptosis, often termed “physiological cell death” (PCD), has been observed in the hippocampus of mice on PD11 following exposure on PD10 to pharmacologic compounds that affect the *N*-methyl-d-aspartate (NMDA) and γ-aminobutyric acid (GABA) systems (Fredriksson et al. 2004). Eriksson’s group has shown previously that neonatal exposure (PD10) to PBDE-99 leads to impaired working memory in hippocampus-related behavioral tests in adulthood (Eriksson et al. 2001). Interestingly, behavioral effects after exposure to NMDA antagonists mimic the effects of PBDE-99 exposure (Fredriksson et al. 2004). However, to our knowledge, no studies have shown an accentuated apoptosis after PBDE-99 treatment. Nonetheless, a recent *in vitro* study showed that PBDE-99 causes apoptotic cell death in an astrocytoma cell line (Madia et al. 2004). Future studies will reveal if PBDE-99 potentiates apoptosis through this pathway in the developing brain *in vivo*.

In conclusion, we present data suggesting that in neonatal mice, early exposure to PBDE-99 may induce cellular stress and that neurodegeneration in combination with aberrant neuroplasticity during this critical stage of brain development may contribute to the late effects in behavior observed in adult mice. It is interesting to note the differences in response to PBDE-99 between the hippocampus and the striatum. Although the general pattern of highly expressed proteins in the two brain parts was strikingly similar, the responses to PBDE-99 were apparently discrete, reflecting the underlying heterogeneity between different brain parts and cell populations. Also, a primary effect in one cell region with axonal projections to other regions would most likely affect the protein expression in the latter regions. By studying molecular effects at the protein level soon after exposure, we have identified proteins that can be evaluated as potential biomarkers that reflect the immediate consequences of early exposure to PBDE-99, and that may provide insights into the processes underlying persistent neurotoxicity.

## Figures and Tables

**Table 1:** Protein identities.

Spot no.	Protein identity^a^	Accession no.^b^	Score^c^	No. of peptides matched	Fold change up (+)/down (−)
Striatum					
1	Albumin-fragment	Q8CG74	89	32	−1.41
2	Albumin 1	Q8C7H3	308	29	−1.24
3	Mortalin	GRP75_MOUSE	213	24	+1.96
4	Methylglyoxalase	LGUL_MOUSE	74	7	−1.33
5	Stathmin	STN1_MOUSE	103	10	−1.30
6	Stathmin	STN1_MOUSE	137	13	−1.21
7	Stathmin	STN1_MOUSE	106	11	−1.26
8	Neuromodulin (Gap-43)	P06837	92	8	+1.31
9	Neuromodulin (Gap-43)	P06837	85	8	+1.25
Hippocampus					
1	Mortalin	GRP75_MOUSE	125	15	+1.52
2	CCT (TCP-1)	TCPB_MOUSE	242	23	+1.21
3	Atp5b	ATPB_MOUSE	219	22	+1.23
4	Macropain	Q8BKU2	125	11	+1.21
5	Gamma enolase	ENOG_MOUSE	229	20	+1.22
6	Gamma enolase	ENOG_MOUSE	256	22	+1.24
7	Alpha enolase	ENOA_MOUSE	216	20	+1.27
8	Laminin receptor 1	P14206	148	12	+1.27
9	Idh3a	Q9D6R2	101	10	+1.58
10	Synuclein, alpha	Q9CXF8	114	9	+1.41

a The identities of 11 spots from striatum and 10 spots from hippocampus, determined by MALDI–ToF–MS peptide mass finger-printing.

b Accession numbers are from the NCBI nonredundant database (http://www.ncbi.nih.org).

c The searches were made using the MASCOT search engine (http://www.matrixscience.com). The peptide masses were matched with the theoretical peptide masses of all proteins in the NCBI nonredundant database. Protein scores > 61 are significant (*p* < 0.05).
